# Traffic-related air pollution and spectacles use in schoolchildren

**DOI:** 10.1371/journal.pone.0167046

**Published:** 2017-04-03

**Authors:** Payam Dadvand, Mark J. Nieuwenhuijsen, Xavier Basagaña, Mar Alvarez-Pedrerol, Albert Dalmau-Bueno, Marta Cirach, Ioar Rivas, Bert Brunekreef, Xavier Querol, Ian G. Morgan, Jordi Sunyer

**Affiliations:** 1 ISGlobal, Centre for Research in Environmental Epidemiology (CREAL), Barcelona, Spain; 2 Pompeu Fabra University, Barcelona, Spain; 3 Ciber on Epidemiology and Public Health (CIBERESP), Madrid, Spain; 4 Institute of Environmental Assessment and Water Research (IDAEA), Spanish National Research Council (CSIC), Barcelona, Spain; 5 Institute for Risk Assessment Sciences, Division Environmental Epidemiology, Utrecht University, Utrecht, The Netherlands; 6 Julius Center for Health Sciences and Primary Care, University Medical Center Utrecht, Utrecht, The Netherlands; 7 ARC Centre of Excellence in Vision Science, Research School of Biology, College of Medicine, Biology and Environment, Australian National University, Canberra, Australia; 8 Department of Preventive Ophthalmology, Zhongshan Ophthalmic Center, Sun Yat-sen University, Guangzhou, China; 9 IMIM-Parc Salut Mar, Spain; McGill University, CANADA

## Abstract

**Purpose:**

To investigate the association between exposure to traffic-related air pollution and use of spectacles (as a surrogate measure for myopia) in schoolchildren.

**Methods:**

We analyzed the impact of exposure to NO_2_ and PM_2.5_ light absorbance at home (predicted by land-use regression models) and exposure to NO_2_ and black carbon (BC) at school (measured by monitoring campaigns) on the use of spectacles in a cohort of 2727 schoolchildren (7–10 years old) in Barcelona (2012–2015). We conducted cross-sectional analyses based on lifelong exposure to air pollution and prevalent cases of spectacles at baseline data collection campaign as well as longitudinal analyses based on incident cases of spectacles use and exposure to air pollution during the three-year period between the baseline and last data collection campaigns. Logistic regression models were developed to quantify the association between spectacles use and each of air pollutants adjusted for relevant covariates.

**Results:**

An interquartile range increase in exposure to NO_2_ and PM_2.5_ absorbance at home was respectively associated with odds ratios (95% confidence intervals (CIs)) for spectacles use of 1.16 (1.03, 1.29) and 1.13 (0.99, 1.28) in cross-sectional analyses and 1.15 (1.00, 1.33) and 1.23 (1.03, 1.46) in longitudinal analyses. Similarly, odds ratio (95% CIs) of spectacles use associated with an interquartile range increase in exposures to NO_2_ and black carbon at school was respectively 1.32 (1.09, 1.59) and 1.13 (0.97, 1.32) in cross-sectional analyses and 1.12 (0.84, 1.50) and 1.27 (1.03, 1.56) in longitudinal analyses. These findings were robust to a range of sensitivity analyses that we conducted.

**Conclusion:**

We observed increased risk of spectacles use associated with exposure to traffic-related air pollution. These findings require further confirmation by future studies applying more refined outcome measures such as quantified visual acuity and separating different types of refractive errors.

## Introduction

Myopia is the most common refractive error of vision, currently affecting about one-fifth of the world’s population (~1.5 billion people) [1–4]. Once considered a purely genetic condition, it is now increasingly recognized as having a multifactorial etiology, with both genetic and environmental factors involved [1, 5–7]. During the past few decades, there has been a notable increase in the global prevalence of myopia, representing an alarming epidemic worldwide [1–4]. Although the reason(s) for this increasing trend are yet to be established, such a rapid increase can be suggestive for a more important contribution of non-genetic and environmental factors in the causation of refractive errors [1, 5, 8].

The increase in the global prevalence of myopia has coincided with the rapid and ongoing increase in the population residing in urban areas where the prevalence of myopia is consistently reported to be higher than rural areas [1, 4, 9–11]. The higher prevalence of myopia in urban areas could suggest that *urban lifestyle* such as more near-work (i.e. tasks such as reading book and working with computer that need sustained gaze on a close object) or less time spent outdoor and/or *urban-related environmental factors* contribute to the pathogenesis of these conditions. Air pollution is the main environmental stressor in urban areas, and is responsible for most of the global burden of disease due to environmental causes [12]. Exposure to traffic-related air pollution is associated with a wide range of adverse health outcomes, with the lungs being one of the most commonly affected organs, mainly because of their constant direct exposure to air pollutants. Similarly, the eyes are directly exposed to air pollution, making them a prime target organ for the adverse effects of such an exposure. In addition to the short-term effects of air pollution on the eye, such as irritation of the ocular surface and its accompanying symptoms and complaints, chronic exposure to air pollution has been associated with long-lasting ocular conditions such as dry eye disease [13] and cataract [14]. Although air pollution could induce myopia through systemic inflammation and oxidative stress (as discussed later in the text), to date no studies have reported on the potential effect of air pollution on the development of myopia.

The aim of this analysis was to investigate the association between exposure to traffic-related air pollution and use of spectacles in schoolchildren. We considered use of spectacles as a surrogate for refractive errors of vision and specifically myopia because when low visual acuity increases during childhood, this is particularly likely to be associated with the onset of myopia [15–17]. Our choice of primary schoolchildren to test our hypothesized association was in line with most studies (e.g. [18–23]) of the environmental determinants of refractive errors that have focused on early years of primary school as a suitable window of exposure because it is a period when environmental factors have greatest opportunity to affect rapidly changing eyes.

## Materials and methods

### Study setting and participants

We undertook this study in Barcelona, Spain, a port city situated on the Northeastern part of the Iberian Peninsula. Air pollution concentrations in Barcelona are among the highest in Europe, partly attributed to high traffic density and large proportion (~50%) of diesel-powered vehicles, relatively low precipitation, high population density (about 16,000/km^2^), and an urban landscape characterized by 5–6 story buildings and narrow streets, which reduces the dispersion of pollutants [24, 25].

This study was based on data collected by the BRain dEvelopment and Air polluTion ultrafine particles in scHool childrEn (BREATHE) project, which aimed to evaluate the impact of air pollution exposure on neurobehavioral development in primary schoolchildren. As described in detail previously [26, 27], of the 416 schools in Barcelona, 40 schools were initially selected to obtain maximum contrast in traffic-related air pollution levels (i.e. NO_2_) of which 39 accepted to participate and were included in the study. Participating schools were similar to the remaining schools in Barcelona in terms of the neighborhood socioeconomic vulnerability index (0.46 versus 0.50, Kruskal—Wallis test p = 0.57) and NO_2_ levels (51.5 versus 50.9 μg/m^3^, Kruskal—Wallis test p = 0.72).

We invited all schoolchildren (n = 5,019) without special needs in the 2^nd^ to 4^th^ grades (7–10 years) of participating schools to participate through letters and/or presentations in schools for parents, of which 2,897 (58%) agreed to participate in BREATHE. All participants had been in the same school for more than six months (and 98% more than one year) before the beginning of the study. All parents or guardians signed the informed consent and the BREATHE project was approved (No. 2010/41221/I) by the Clinical Research Ethical Committee of the Parc de Salut MAR, Barcelona, Spain.

### Outcome and covariate data

We considered the use of spectacles as a surrogate for myopia and applied it as a binary (yes/no) outcome variable. Data on the use of spectacles reported by parents were collected twice: once in the first (i.e. baseline) data collection campaign during 2012 and once during the last data collection campaign during 2015. Sociodemographic data including child’s sex and age and parental ethnicity and indicators of socioeconomic status such as educational achievement and employment status together with data on pregnancy period and childhood were obtained from parents through questionnaires.

### Air pollution exposure

We assessed exposure to NO_2_, and particulate matter with aerodynamic diameter ≤ 2.5 μm (PM_2.5_) light absorption (hereafter referred to as *PM*_*2*.*5*_ *absorbance*, a proxy for black carbon (BC)) at residential addresses and to NO_2_ and BC at schools. These pollutants have been used extensively as markers of air pollution generated by traffic.

#### Residential air pollution levels

We utilized an established spatiotemporal exposure assessment framework based on temporally-adjusted spatial estimates of air pollutant levels by land use regression (LUR) models developed as part of the European Study of Cohorts for Air Pollution Effects (ESCAPE) [28, 29]. These models could predict 72% and 83% of variation in annual (2009) levels of NO_2_ and PM_2.5_ absorbance, respectively, across the Barcelona [30]. By temporally adjusting (ratio method) the LUR spatial estimates, we were able to predict the ambient pollutant levels at the geocoded home address of each study participant for the periods between 1) her/his birth and baseline data collection campaign (hereafter referred to as *lifelong exposure*) and 2) between the baseline and last data collection campaigns (hereafter referred to as *prospective exposure*). Further details of our applied spatiotemporal exposure assessment framework and LUR models have been reported before [28–30]. As part of BREATHE questionnaire, the participants were asked to report their current address together with their previous addresses and the period they spent in each address.

#### School air pollution levels

As described in detail previously [26, 31], air pollution levels at each BREATHE school were measured twice during one-week campaigns separated by six months, once during the warm and once during the cold seasons of the year 2012. Air samples were collected in a classroom (i.e. indoor) at a height between 0.7 and 1.5 m above floor level, which is at the eye level of pupils aged 7–9 years and is also the height at which they would usually inhale. Weekly averaged NO_2_ concentrations were measured by Gradko Environmental passive dosimeters. BC concentrations were measured using the MicroAeth AE51 (AethLabs). Considering that the air pollution sampling in different schools were conducted during different weeks in each campaign period, we deseasonalized the monitored air pollution levels using the levels (during the corresponding sampling week for each school) measured by a background air pollution monitoring station in Barcelona to remove temporal fluctuation in background levels from our analyses using a method that has been reported previously [31].

### Data analysis

We used cross-sectional and longitudinal frameworks to analyze the association between air pollution exposure and spectacles use. For the cross-sectional analyses, we applied spectacles use at baseline (i.e. prevalent spectacles use) as the outcome variable and *lifelong exposure* to residential air pollution as the main exposure variable. The longitudinal framework was based on the incidence of spectacles use and exposure to air pollution during the three-year period (2012–2015) between the baseline and last data collection campaigns. Accordingly, we developed a binary variable indicating whether the participant started to use spectacles during this period and we used this variable as the outcome variable together with *prospective exposure* to residential air pollution as the main exposure variable for the longitudinal analyses. For the exposure to air pollution at schools, we used the annual estimate (2009) for both cross-sectional and longitudinal analyses.

Because of the multilevel nature of the data (children at schools), we used logistic mixed effects models with prevalent/incident spectacles use (one at a time) as the outcome variable, estimates of lifelong/prospective exposure to each pollutant at home and school (one at a time) as the fixed effect predictor, and school as random effect. For the longitudinal analyses, we excluded those participants using spectacles at baseline since they could not be considered to be at the risk of using spectacles during the course of the follow-up. The analyses were adjusted for a number of covariates identified *a priori*: age (at the time of the baseline data collection for the cross-sectional analyses and the time of the last data collection campaign for the longitudinal analyses), sex, paternal and maternal ethnicities (European or non-European), prematurity (yes/no) [32–34], child’s exposure to environmental tobacco smoke (yes/no) [34, 35], child’s average screen time per week, child annual total time (hours) of playing in green spaces, and indicators of socioeconomic status (SES) at both individual and area levels. In particular, we used weekly screen time as a surrogate for ‘near-work’ and child annual total time of playing in green spaces as a surrogate for outdoor activity which have been associated with the risk of myopia [1]. Screen time and green space playing time were reported by parents as the average time (separately for working days and weekends) the child would spend on watching TV or playing game on videogame console or computer and the average time the child would spend playing in green spaces separately for the working days and weekends during school period and summer holidays. Paternal and maternal educational achievements (primary school, secondary school, or university) was used as the indicator of individual-level SES and Urban Vulnerability Index [36], a measure of neighborhood SES at the census tract (median area of 0.08 km^2^ for the study region) was applied as the indicator of area-level SES. The odds ratios (ORs) were reported for an interquartile range (IQR) increase in each pollutant based on all study participants separately for cross-sectional and longitudinal analyses.

## Results

Of 2727 (94.1%) BREATHE participants with available data on spectacles use who were included in the cross-sectional analyses, 359 (13.2%) used glasses at baseline. As presented in Table 1, those participants using spectacles were more likely to be girls (p = 0.07), older (p<0.01), and spending less time playing outdoors in green spaces (p = 0.06).

**Table 1:** Description^a^ of characteristics of the study participants.

Variables^a^	Participants without spectacles(n = 2,368)	Participants with spectacles (n = 359)	Prevalence of spectacles use	p-value^b^
**Child age (Years)**	8.5 (1.4)	8.7 (1.6)	-	<0.01
**Child sex**				0.07
Female	1,163 (49.1%)	194 (54.0%)	14.3%	
Male	1.203 (50.8%)	163 (45.4%)	11.9%	
Missing	2 (0.1%)	2 (0.6%)		
**Maternal educational achievement**				
No or primary education	297 (12.5%)	48 (13.4%)	13.9%	0.11
Secondary education	660 (27.9%)	117 (32.6%)	15.1%	
University	1.396 (59.0%)	191 (53.2%)	12.0%	
Missing	15 (0.6%)	3 (0.8%)		
**Paternal educational achievement**				0.14
No or primary education	366 (15.5%)	53 (14.8%)	12.7%	
Secondary education	716 (30.2%)	125 (34.8%)	14.9%	
University	1,241 (52.4%)	169 (47.1%)	12.0%	
Missing	45 (1.9%)	12 (3.3%)		
**Maternal ethnicity**				0.20
European	2,082 (87.9%)	307 (85.5%)	12.9%	
Non-European	286 (12.1%)	52 (14.5%)	15.4%	
**Paternal ethnicity**				0.78
European	2,064 (87.2%)	311 (86.6%)	13.1%	
Non-European	304 (12.8%)	48 (13.4%)	13.6%	
**Prematurity**				0.44
Yes	170 (7.2%)	29 (8.1%)	14.6%	
No	2,113 (89.2%)	307 (85.5%)	12.7%	
Missing	85 (3.6%)	23 (6.4%)		
**Exposure to environmental tobacco exposure**				
Yes	282 (11.9%)	54 (15.0%)	16.1%	0.10
No	2,064 (87.2%)	303 (84.4%)	12.8%	
Missing	22 (0.9%)	2 (0.6%)		
**Total green space playing time (hours per year)**	486 (511)	442 (528)	-	0.06
**Total screen time (hours per week)**	4.5 (3)	4 (3)	-	0.19

^a^ For continuous variables, median (IQR) and for categorical variables count (percentage) of each category has been reported.

^b^ p-value of chi-squared test for categorical variables and Mann—Whitney U test for continuous variables.

Of BREATHE participants included in the cross-sectional analyses, 1812 (66.5%) were followed till the last data collection campaign and included in the longitudinal analyses. The last data collection campaign identified 155 incident cases of spectacles use (i.e. 155 BREATHE participants started to use spectacles during our three-year follow-up period between the first and last data collection campaigns). Compared to participants included in the cross-sectional analyses, those included in the longitudinal analyses were younger (p<0.01) at baseline (S1 Table) which was expected because older children were supposed to finish primary school and move to high school before our last data collection campaign (This was the main reason for lost to follow-up in our study). The participants included in the longitudinal analyses also reported less screen time (p<0.01) and were more likely to be of European descent (p<0.01).

The Spearman’s correlation coefficients between different exposures are presented in S2 Table. While levels of different pollutants at school and at home were strongly correlated, the correlation between school and home levels of each pollutant was weak to moderate.

### Cross-sectional analyses

An IQR increase in NO_2_ level at home was associated with 16% (95% confidence intervals (CIs): 3%, 29%) increase in spectacles use (Table 2). Similarly, we observed an increase in the risk of using spectacles associated with one-IQR increase in exposure to PM_2.5_ absorbance at home but the association was marginally statistically significant.

**Table 2:** Median (InterQuartile Range, IQR) of air polltants and adjusted^a^ odds ratio (95% confidence intervals) of the use of spectacles associated with one Inter-Quartile Range (IQR) increase in exposure to each pollutant.

Air Pollutant	Cross-sectional analyses (N = 2727)			Longitudinal analyses (N = 1812)		
	Median (IQR)	OR(95% CI)	p-value	Median (IQR)	OR(95% CI)	p-value
**Home**						
NO_2_ (μg/m^3^)	50.3 (14.8)	1.16 (1.03, 1.29)	0.01	67.9 (19.6)	1.15 (1.00, 1.33)	0.06
PM_2.5_ Absorbance (10^−5^/m^3^)	2.6 (0.8)	1.13 (0.99, 1.28)	0.06	2.3 (0.8)	1.23 (1.03, 1.46)	0.02
**School**						
NO_2_ (μg/m^3^)	29.8 (21.6)	1.32 (1.09, 1.59)	<0.01	29.8 (21.6)	1.12 (0.84, 1.50)	0.45
BC (μg/m^3^)	1.4 (0.9)	1.13 (0.97, 1.32)	0.13	1.4 (0.9)	1.27 (1.03, 1.56)	0.02

^a^ Adjusted for age, sex, paternal and maternal ethnicities, paternal and maternal educational attainment, prematurity, child’s exposure to environmental tobacco smoke, child’s average screen time per week, child annual total time (hours) of playing in green spaces, and neighborhood socioeconomic status.

An IQR increase in NO_2_ level at school was associated with 32% (95% CIs: 9%, 59%) increase in the risk of wearing spectacles. BC exposure at school was also associated with spectacles use but the association did not attain statistical significance.

### Longitudinal analyses

For residential NO_2_ exposure we observed identical association in terms of direction and strength with that of the cross-sectional analysis (Table 2); however, as expected (because of smaller sample size), the 95% confidence intervals (CI) were wider and include one (p-value = 0.06). The longitudinal association for residential exposure to PM_2.5_ absorbance became stronger compared to that of cross-sectional analyses and attained statistical significance (Table 2). Similarly, the association for school BC exposure was stronger and statistically significant in the longitudinal analyses (Table 2). On the other hand, the association for NO_2_ exposure at school became weaker and lost its statistical significant in longitudinal analyses.

The estimates for 10 unit increase in NO_2_ (μg/m^3^) and one unit increase in PM_2.5_ Absorbance (10^−5^/m^3^) and Black Carbon (μg/m^3^) for cross-sectional and longitudinal analyses are presented in S3 Table.

### Sensitivity analyses

Further adjustment of analyses for maternal smoking during pregnancy [33, 37, 38], breastfeeding [34], neighborhood socioeconomic status (Urban Vulnerability Index) of the school, parental employment status (unemployed, employee, or self-employed), parental marital status, and child’s height [34] did not change our findings notably (Data not shown). However, after adjustment of cross-sectional analyses for parental employment status, the association for residential PM_2.5_ absorbance became stronger and attained statistical significance (OR: 1.13, 95% CIs: 1.00, 1.29). Limiting the cross-sectional analyses of residential air pollution to those who had not moved since birth (n = 1689, 61.9%) did not result in a considerable change in direction and strength of the associations, but, as expected, the confidence intervals for NO_2_ became wider and the association became nearly statistically significant (OR: 1.14, 95% CI: 0.99, 1.32, p-value = 0.07). Additionally, we did not observe any statistically significant effect modification by child’s sex, maternal education or neighborhood SES for our associations.

## Discussion

### Interpretation of results

To our knowledge, this is the first study to evaluate the association between air pollution exposure and the use of spectacles (a marker of refractive errors of vision and specifically myopia). We observed an increased likelihood of spectacles use associated with higher exposure to traffic-related air pollutants that were generally consistent for exposures at home and at school and in cross-sectional and longitudinal analytical frameworks. These findings were also robust against a range of sensitivity analyses that we conducted.

Further adjustment of our analyses for SES indicators other than educational attainments of parents and residential neighborhood SES such as parental employment status, marital status and school neighborhood SES did not result in a notable change in our findings. Moreover, the indicators of SES (parental educational attainment and neighborhood SES) were not associated with the risk of spectacles use (Table 1). These observations could suggest that our analyses were not likely to have been influenced by residual SES confounding.

While the results of residential exposure to air pollution were consistent in cross-sectional and longitudinal analyses, for air pollution exposure at school we observed a difference between findings of these analytical frameworks. The findings for the cross-sectional analyses of school exposures need to be interpreted with caution because most of the study participants were recruited in their first years of primary schools, thus for them the length of exposure to school air pollution before reporting spectacles use at baseline could have been too short to be able to induce refractive errors. On the other hand, the consistency of our findings (in terms of direction and strength of associations) for the exposure to air pollution at school in the longitudinal analyses with those of residential exposure to air pollution in both cross-sectional and longitudinal analyses could offer us more confidence about these findings.

### Available evidence and potential underlying mechanisms

We are not aware of any previous epidemiological studies on our investigated association between air pollution and refractive errors; therefore, it is not possible to compare our findings with those of others. There are also no available animal model or *in vitro* or *in vivo* studies evaluating the direct link between exposure to air pollution and refractive errors of vision. However, our findings are consistent with a number of previous observations. A recent study has reported a higher risk of “near visual difficulty” (defined as difficulty in seeing and recognizing an object at arm’s length) associated with having a cooking stove in the same room as sleeping area which could be an indicator of indoor air pollution [39].

Local and systemic inflammation and oxidative stress are the most established mechanisms for the adverse health effects of air pollution. Systemic inflammatory diseases often affect different parts of the eyes, including the sclera, cornea, vitreous, and retina, resulting in intraocular inflammatory conditions such as uveitis and retinal vasculitis [40]. Similarly, systemic inflammation induced by long-term exposure to air pollution can prompt changes in retinal microvasculature, including narrowing of arteriolar diameters and widening of venular diameters, which in turn have been associated with arteriolar damage, endothelial dysfunction, and intraocular inflammation [41–45]. The latter can induce myopia in the eyes by affecting the optical power of the lens [46] and/or impairing retinal neuroactivity [47], which regulates the axial growth of eyes early in life. Inadequate or excessive axial growth of eyes relative to the ocular refractive power is one of the known causes of myopia. In addition to inducing systemic inflammation, exposure to air pollution generates local inflammation on the ocular surface [13, 48]. Animal studies have shown that eye surface inflammation can infiltrate into the eye [49], resulting in retinal inflammation [50], which in turn can affect its neuroactivity and regulation of axial length growth.

In addition to inflammation, air pollution induces oxidative stress in the eyes, which has been reported to be involved in a number of conditions such as cataract, uveitis, age-related macular degeneration, glaucoma, and various types of retinopathy [51, 52]. Oxidative stress has been shown to impair the release of dopamine from retinal cells [53], which plays a critical role in regulating the axial growth of the eye [1, 8, 54]. Therefore, oxidative stress-related impairment of dopamine release from retinal cells could be one explanation for the impact of air pollution on myopia.

Furthermore, air pollution has been implicated as a risk factor for dry eye disease by inducing instability of tear film, ocular surface inflammation, epithelial differentiation and hyperplasia of goblets cells [13, 48]. Dry eye disease has been shown to result in reduction in corneal thickness [55] and irregularities in corneal surface [56] which can ultimately lead to impaired visual acuity [57, 58]. Dry eye disease has also been reported to increase oxidative stress in conjunctival epithelium [59] which could contribute to the aforementioned oxidative stress pathway.

### Limitations of study

The generalizability of our findings might have been affected by selection bias in that those participants participated in BREATHE might be different from those not participated with respect to SES. The Urban Vulnerability Index of the schools was not associated with school participation rate (Spearman’s correlation coefficient = -0.11, p-value = 0.51); this might suggest that the SES was less likely to be a major predictor of participating in the study. A part from non-participation, we had additional loss to follow-up from baseline to the end of the study. Those participants dropped out of the study during the follow-up period were different from those followed until the end of the study in terms of age, screen time and ethnicity, and maternal education which could have introduced selection bias in our findings. As the differing characteristics are not expected to be associated to residential air pollution levels, we do not expect this loss to follow-up to bias the results. We used eyeglasses as a surrogate for myopia. However, the use of eyeglasses by our participants could also have been due to other refractive errors such as hyperopia and astigmatism that have different pathogenesis. This could be more relevant for our cross-sectional analyses based on the prevalent eyeglasses use. On the other hand, considering the age of our study participants, we expect that the incident reduction in visual acuity during the course of our longitudinal study is more strongly associated with myopia than other refractive errors [15–17]. Therefore, the findings of our longitudinal analyses were more likely to be relevant to myopia compared to those of cross-sectional analyses. Moreover, children with less severe refractive problems who did not use spectacles were not considered as having refractive errors in our study which could have biased our findings towards null. Likewise, we did not obtain information on use of contact lenses and the resulting outcome misclassification could have biased our estimates towards null. Furthermore, we did not have data on refractive errors in parents enabling us to address the genetic contribution in our analyses. However, we do not have any reason to assume a differential exposure to air pollution for children of parents with and without refractive error. Moreover, by temporally adjusting the LUR spatial estimates of pollutant levels, we effectively assumed that the city spatial surface and the spatial distribution of pollutants remained unchanged over the study period. Previous studies in Europe have shown the stability of these spatial contrasts over a long period [60, 61]. Moreover, we are not aware of any major change in traffic flow, land use, or emissions profiles occurred between the year of LUR model construction and the study period. Accordingly, we observed a strong correlation (Spearman’s correlation coefficient of 0.81) between modeled NO_2_ levels at school using LUR models and measured NO_2_ levels at schools during BREATHE campaigns, assuring us about the long-term validity the assigned home exposure levels.

## Conclusions

We found an increase in the likelihood of myopia (as surrogated by spectacles use) in association with exposure to traffic-related air pollution at home and at school. Because of the aforementioned limitations, this study might not be able to establish a causal link; however, considering the consistent pattern of our observed associations for school and residential exposures while they were weakly correlated, the consistency of our findings based on cross-sectional and longitudinal analyses, and the robustness of these associations to several sensitivity analyses, we are convinced that our findings merits further investigation.

Currently, about half of the world population reside in cities, and it is predicted that by 2030 around 70% of global population will live in urban areas [62] where myopia is more prevalent. According to a recently published report by the WHO, most of the world’s cities (mainly in developing countries) are currently in breach of its guidelines on air pollution levels [63]. Uncorrected refractive errors are a major contributor to the global burden of disease accounting for more than 11 million disability-adjusted life-years (DALYs) [64]. Taking into account such a considerable burden of refractive errors with the ongoing rise in their prevalence worldwide, an adverse impact of air pollution on these conditions, if established by future studies, offers policymakers an evidence base for developing policies and implementing targeted interventions in order to slow down and ideally reverse the current ongoing rise in prevalence of eye refractive errors. Such an impact could also open a whole new area in our understanding of the causes of eye refractive errors in general and myopia in particular as well as adverse health effects of air pollution which are of great importance for research community in various disciplines. Further animal, in vivo, and in vitro studies are required to elucidate potential pathways underling such an impact, if any. We advise future epidemiological studies to apply more refined outcome measures such as quantified visual acuity and to separate different types of refractive errors.

## Supporting information

S1 Table Description of characteristics of the study participants included in cross-sectional and longitudinal analyses. (DOCX)

S2 Table Spearman’s correlation coefficient between estimates of air pollution for the cross-sectional analyses. (DOCX)

S3 Table Adjusted odds ratio (95% confidence intervals) of the use of spectacles associated with 10 unit increase in NO_2_ (μg/m^3^) and one unit increase in PM_2.5_ Absorbance (10^−5^/m^3^) and Black Carbon (μg/m^3^). (DOCX)
