# Supplementary material for: Traffic-related air pollution and spectacles use in schoolchildren
Source: PLoS One. 2017 Apr 3;12(4):e0167046. doi: 10.1371/journal.pone.0167046 (PMC5378327; doi:10.1371/journal.pone.0167046)
Supplement: S1 Table — (DOCX) [file pone.0167046.s001.docx]

**S1 Table.** Description^a^ of characteristics of the study participants included in cross-sectional and longitudinal analyses.

| **Variables** | **Participants in  cross-sectional analyses (n=2727)** | **Participants in  longitudinal analyses (n=1812)** | **p-value^b^** |
| --- | --- | --- | --- |
| **Child age (Years) at baseline** | 8.5 (1.5) | 8.3 (1.2) | <0.01 |
| **Child sex** |  |  | 0.88 |
| Female | 1,357 (49.8%) | 875 (48.3%) |  |
| Male | 1,366 (50.1%) | 889 (49.1%) |  |
| Missing | 4 (.1%) | 48 (2.6%) |  |
| **Maternal educational achievement** |  |  | 0.10 |
| No or primary education | 345 (12.7%) | 195 (10.8%) |  |
| Secondary education | 777 (28.5%) | 479 (26.4%) |  |
| University | 1,587 (58.2%) | 1,081 (59.7%) |  |
| Missing | 18 (.7%) | 57 (3.1%) |  |
| **Paternal educational achievement** |  |  | 0.06 |
| No or primary education | 419 (15.4%) | 232 (12.8%) |  |
| Secondary education | 841 (30.8%) | 539 (29.7%) |  |
| University | 1,410 (51.7%) | 969 (53.5%) |  |
| Missing | 57 (2.1%) | 72 (4%) |  |
| **Maternal ethnicity** |  |  | <0.01 |
| European | 2,389 (87.6%) | 1,649 (91%) |  |
| Non-European | 338 (12.4%) | 163 (9%) |  |
| **Paternal ethnicity** |  |  | <0.01 |
| European | 2,375 (87.1%) | 1,644 (90.7%) |  |
| Non-European | 352 (12.9%) | 168 (9.3%) |  |
| **Prematurity** |  |  | 0.67 |
| Yes | 199 (7.3%) | 136 (7.5%) |  |
| No | 2,420 (88.7%) | 1,572 (86.8%) |  |
| Missing | 57 (2.1%) | 104 (5.7%) |  |
| **Exposure to environmental tobacco smoke** |  |  | 0.38 |
| Yes | 336 (12.3%) | 202 (11.1%) |  |
| No | 2,367 (86.8%) | 1,547 (85.4%) |  |
| Missing | 24 (.9%) | 63 (3.5%) |  |
| **Total screen time (hours) per week)** | 4.5 (3) | 4 (3) | 0.02 |

^a^ For continuous variables, median (IQR) and for categorical variables count (percentage) of each category has been reported.

^b^ p-value of chi-squared test for categorical variables and Mann–Whitney U test for continuous variables.
