# Supplementary material for: Traffic-related air pollution and spectacles use in schoolchildren
Source: PLoS One. 2017 Apr 3;12(4):e0167046. doi: 10.1371/journal.pone.0167046 (PMC5378327; doi:10.1371/journal.pone.0167046)
Supplement: S2 Table — (DOCX) [file pone.0167046.s002.docx]

**S2 Table.** Spearman’s correlation coefficient between estimates of air pollution for the cross-sectional analyses.

|  | **Residential NO_2_** | **Residential PM_2.5_ Absorbance** | **School NO_2_** | **School BC** |
| --- | --- | --- | --- | --- |
| **Residential NO_2_** | 1 |  |  |  |
| **Residential PM_2.5_ Absorbance** | 0.91* | 1 |  |  |
| **School NO_2_** | 0.37* | 0.34* | 1 |  |
| **School Black Carbon (BC)** | 0.28* | 0.27* | 0.74* | 1 |

* p-value <0.05
