# Supplementary material for: Traffic-related air pollution and spectacles use in schoolchildren
Source: PLoS One. 2017 Apr 3;12(4):e0167046. doi: 10.1371/journal.pone.0167046 (PMC5378327; doi:10.1371/journal.pone.0167046)
Supplement: S3 Table — (DOCX) [file pone.0167046.s003.docx]

**S3 Table.** Adjusted^a^ odds ratio (95% confidence intervals) of the use of spectacles associated with 10 unit increase in NO_2_ (µg/m^3^) and one unit increase in PM_2.5_ Absorbance (10^−5^/m^3^) and Black Carbon (µg/m^3^).

| **Air Pollutant** | **Cross-sectional analyses (N=2727)** | |  | **Longitudinal analyses (N=1812)** | |
| --- | --- | --- | --- | --- | --- |
|  | OR(95% CI) | p-value |  | OR(95% CI) | p-value |
| **Home** |  |  |  |  |  |
| NO_2_ | 1.10 (1.02, 1.18) | 0.02 |  | 1.08 (1.00, 1.16) | 0.06 |
| PM_2.5_ Absorbance | 1.16 (0.99, 1.35) | 0.06 |  | 1.30 (1.04, 1.62) | 0.02 |
| **School** |  |  |  |  |  |
| NO_2_ | 1.14 (1.04, 1.25) | <0.01 |  | 1.05 (0.92, 1.21) | 0.45 |
| BC | 1.15 (0.96, 1.39) | 0.13 |  | 1.32 (1.04, 1.67) | 0.02 |

^a^ Adjusted for age, sex, paternal and maternal ethnicities, paternal and maternal educational attainment, prematurity, child’s exposure to environmental tobacco smoke, child’s average screen time per week, child annual total time (hours) of playing in green spaces, and neighborhood socioeconomic status.
